# Evaluation of LiangXue JieDu Therapy in Combination With Western Medicine for Acute-On-Chronic Liver Failure: A Systematic Review and meta-Analysis

**DOI:** 10.3389/fphar.2022.905215

**Published:** 2022-07-12

**Authors:** Ke Shi, Qun Zhang, Jie Hou, Yi Zhang, Yufei Bi, Xianbo Wang

**Affiliations:** Center of Integrative Medicine, Beijing Ditan Hospital, Capital Medical University, Beijing, China

**Keywords:** LiangXue JieDu, traditional Chinese medicine, meta-analysis, actue-on-chronic liver failure, randomized controlled trials

## Abstract

**Objectives:** To assess the efficacy of LiangXue JieDu (LXJD) therapy in combination with Western medicine (WM) for acute-on-chronic liver failure (ACLF).

**Methods:** Articles on randomized controlled trials of LXJD therapy for ACLF were obtained from PubMed, Embase, Cochrane Library, Web of Science, Chinese National Knowledge Infrastructure, VIP, Wanfang, and China Biology Medicine databases, with the search range from database inception to March 2022. We evaluated the quality of data from these articles using the Cochrane risk-of-bias tool. Evaluation indicators were total effective rate, mortality rate, complications, liver and coagulation function, and Traditional Chinese medicine (TCM) syndrome score. We then calculated the risk ratio (RR) for dichotomous variables and mean difference (MD) for continuous variables with a 95% confidence interval (CI).

**Results:** The meta-analysis included 18 studies with moderate quality and totaling 1,609 patients. Compared with WM alone, LXJD therapy plus WM improved total effective rate [RR = 1.34, 95% CI: (1.24, 1.45)], while reducing mortality rate [RR = 0.54, 95% CI: (0.42, 0.70)] and complications [RR = 0.43, 95% CI: (0.26, 0.71)]. The combined treatment also improved prothrombin activity [MD = 1.30, 95% CI: (1.02, 1.59)], prothrombin time [MD = −0.90, 95% CI: (−1.40, −0.39)], international normalized ratio [MD = −0.59, 95% CI: (−0.93, −0.25)], alanine aminotransferase [MD = −0.92, 95% CI: (−1.30, −0.55)], aspartate aminotransferase [MD = −0.57, 95% CI: (−0.93, −0.21)], total bilirubin [MD = −1.07, 95% CI: (−1.38, −0.76)], and TCM syndrome score [MD = −1.70; 95% CI: (−2.03, −1.37)].

**Conclusions:** This study suggests that LXJD therapy plus WM can significantly improves ACLF clinical symptoms and short-term outcomes. However, more high-quality trials are required to confirm the efficacy of LXJD therapy.

## 1 Introduction

Acute-on-chronic liver failure (ACLF) refers to the acute decompensation of chronic liver disease, with jaundice, coagulopathy, ascites, and hepatic encephalopathy as the main clinical manifestations (Wu et al., 2018). In China, the main etiology of ACLF is associated with hepatitis B virus infection (Bernal et al., 2015). A major health problem, ACLF results in high mortality rate and severe multi-organ damage (Li et al., 2021). Currently, effective drugs for ACLF are lacking and treatment mainly involves artificial livers, liver transplantation, and other symptomatic treatments (Sarin and Choudhury., 2016). However, a shortage in donor livers limits widespread implementation of transplants, and overall clinical efficacy has been unsatisfactory (Sarin and Choudhury, 2016). Therefore, novel treatment methods for ACLF are urgently needed.

Traditional Chinese medicine (TCM) has been used for centuries to treat liver disease. TCM is clinically effective for promoting lowering jaundice, endotoxins, and inflammation, while enhancing liver regeneration (Cai et al., 2020; Xu et al., 2020). TCM categorizes ACLF as “jaundice,” and its basic pathogenesis is concentrated in “poison, heat, dampness, and stasis” (Shi et al., 2021). Syndromes associated with heat, poison, and stasis such as ACLF are commonly treated with the cool blood detoxification method (“LiangXue JieDu” in Chinese, abbreviated to LXJD) (Liu et al., 2014; Zhang, 2021). TCM theory postulates that heat, toxins, and blood stasis are pathological products and pathogenic factors. Both stasis and heat negatively affect liver function and cause complications. In principle, LXJD lowers heat, detoxifies, cools blood, and activates blood circulation, resulting in unobstructed Qi and blood flow; as a result, liver damage and disease progression are prevented (China Association of Chinese Medicine, 2019). Several clinical studies have recently evaluated the combined effects of LXJD therapy and Western medicine (WM) on ACLF. For example, a previous study found that detoxification and stasis-resolving granules are beneficial for improving jaundice and alleviating various other symptoms (Wang et al., 2014). Our previous study also reported that Jiedu Liangxue Jianpi prescriptions had a positive effect on ACLF treatment (Shi et al., 2021). However, the efficacy of LXJD therapy has not yet been systematically evaluated.

Accordingly, this study performed a meta-analysis of randomized controlled trials (RCTs) to assess the efficacy of LXJD combined with WM in treating ACLF.

## 2 Materials and Methods

### 2.1 Search Strategy

The study was performed according to the Preferred Reporting Items for Systematic Review and Meta-Analysis (PRISMA) guidelines (Cumpston et al., 2019). Two researchers (KS and JH) separately searched eight databases (PubMed, Embase, Cochrane Library, Web of Science, Chinese National Knowledge Infrastructure, Wanfang, VIP, and China Biology Medicine) from their inception until March 2022. Search languages were Chinese and English. We conducted manual retrieval and secondary searches to ensure comprehensive literature retrieval. Table 1 shows the PubMed search strategy as an example.

**TABLE 1:** Search strategy.

#1 acute-on-chronic liver failure [title/topic]
#2 ACLF [title/topic]
#3 liver failure [title/topic]
#4 Blood cooling detoxification method [title/topic]
#5 Blood cooling [title/topic]
#6 detoxification [title/topic]
#7 LiangXue JieDu therapy [title/topic]
#8 LiangXue [title/topic]
#9 JieDu [title/topic]
#10 #1 OR #2 OR #3 OR #4 OR #5 OR #6 OR #7 OR #8 OR#9
#11 Randomized controlled trials [title/topic]
#12 Random [title/topic]
#13 #10 OR #11 OR #12

### 2.2 Inclusion and Exclusion Criteria

Studies were included if: 1) patients were diagnosed with ACLF according to consensus recommendations of the Asian Pacific Association for the Study of the Liver (APASL) (Sarin et al., 2019); 2) they were RCTs; 3) treatment involved LXJD therapy plus WM; 4) patients were classified as having heat-toxin-stasis syndrome; 5) at least one of the following indicators were reported: total effective rate, mortality rate, complication, alanine aminotransferase (ALT), aspartate aminotransferase (AST), total bilirubin (TBIL), albumin (ALB), prothrombin activity (PTA), prothrombin time (PT), international normalized ratio (INR), and TCM syndrome score. Primary outcomes were total effective rate and mortality rate, whereas secondary outcomes were complication rate, liver function, coagulation function, and TCM syndrome score.

Exclusion criteria were as follows: 1) duplicated or redundant study; 2) reviews, animal experiments, and non-RCTs; 3) non-LXJD therapy or LXJD combined with other TCM therapies; 4) acute, subacute, or chronic liver failure; 5) incomplete data.

### 2.3 Data Extraction and Quality Assessment

Two researchers (KS and JH) independently performed the literature search and extracted data based on inclusion and exclusion criteria. Data extraction included title, author name, publication date, sample size, gender, age, intervention measures, course of treatment, and observed outcome indicators. If the two researchers disagreed, a third party was consulted to reach a resolution (QZ and YFB).

Literature quality was evaluated using the Cochrane Collaboration’s tool, accounting for seven sources of bias: random-sequence generation, allocation concealment, blinding of investigators and participants, blinding of outcome evaluation, incomplete outcome data, selective reporting, and other. Reports in line with quality evaluation criteria were categorized as low risk; otherwise, they were considered high risk. Studies without sufficient information for assessment were labeled as unclear risk.

### 2.4 Statistical Analysis

All data analyses were performed in Stata 16.0 (Stata Corp, College Station, TX) and R (version 4.0.5, The R Foundation, Vienna, Austria). Risk ratio (RR) and 95% confidence interval (CI) were used for analyzing dichotomous variables. Continuous variables were analyzed using mean difference (MD) and 95% CI. Effect models were selected based on *I* ^*2*^ and *p*-values. A random-effects model was used when *I* ^*2*^ > 50% or *p* < 0.1; otherwise, a fixed-effects model was applied. Sensitivity analysis was conducted to assess the stability of results after removing individual studies. A funnel plot was generated to evaluate potential publication bias.

## 3 Results

### 3.1 Literature Search and Patients’ Characteristics

**FIGURE 1:**
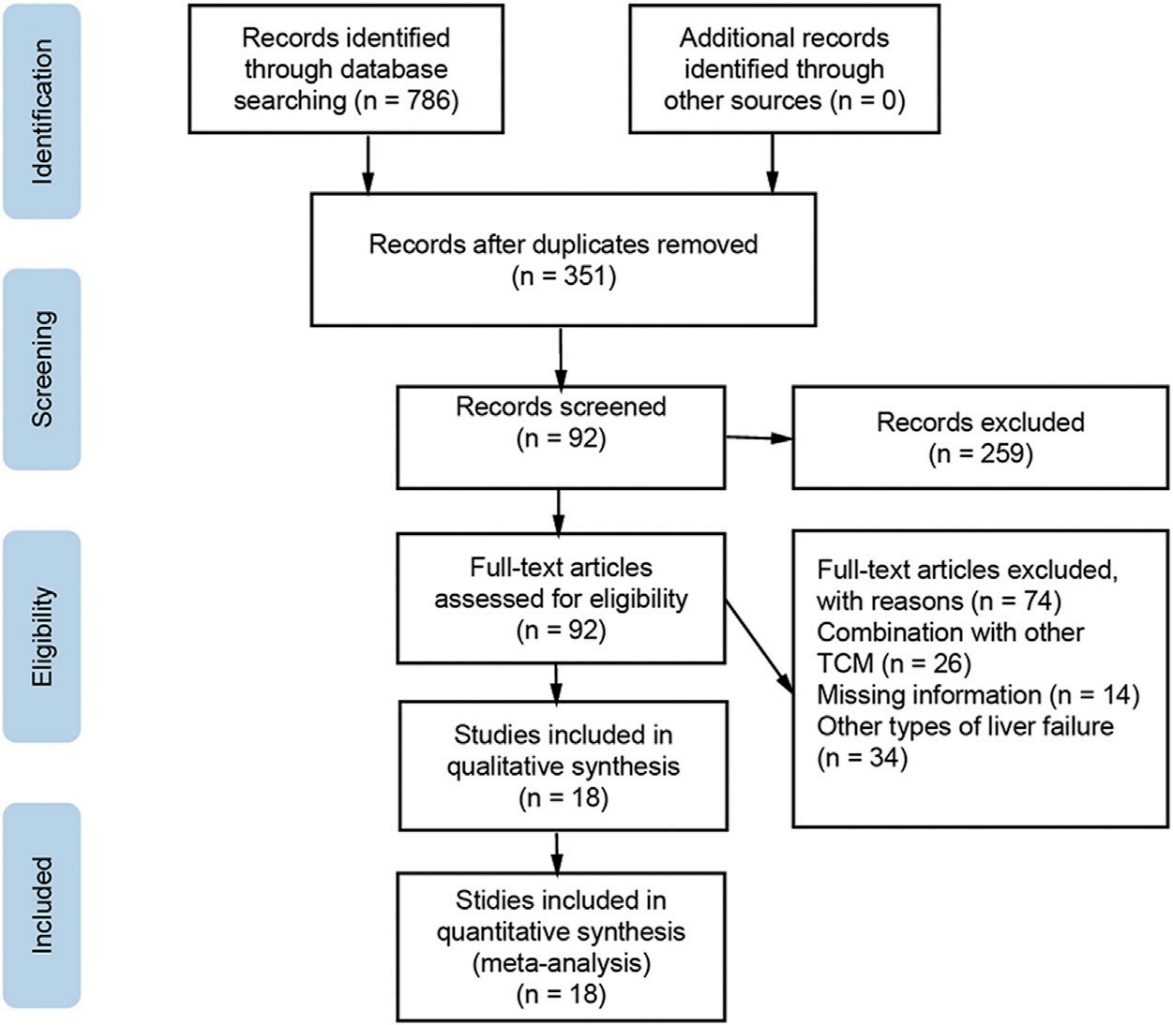
Flowchart of literature selection and selection.

The literature search initially yielded 786 articles, and 435 duplicate studies were excluded. Another 259 articles were excluded because they were reviews, animal experiments, or non-RCTs. The remaining 92 articles were downloaded for full text review. Of these, 74 studies were further excluded because they lacked primary data, combined WM with other TCM, or did not investigate ACLF. The final meta-analysis included 18 studies and 1,609 patients (Liu et al., 2014; Wang et al., 2014; Liu et al., 2015; Duan et al., 2016; Sun et al., 2016; Xiao et al., 2016; Zhao, 2016; Dang et al., 2017; Liu and Bai, 2017; Lou, 2017; Pang and Yang, 2017; Chen et al., 2018; Shi et al., 2018; Yin and Wen, 2018; Zhou et al., 2018; Shi et al., 2021; Zhang, 2021; Huang et al., 2022). Inclusion and exclusion procedures are shown in Figure 1. Features of included studies are presented in Table 2.

**TABLE 2:** Characteristics of included studies.

Author, year	Cases		Age	Gender		Course of disease range	Intervention		Duration/follow up	Outcome measures^a^
	T	C		Male	Female		T	C		
Liu et al., (2014)	64/41		T: 43 ± 21.02	97/8		T: 15.41 ± 3.12	LiangXue JieDu Decoction + WM	WM	8 W/48 W	1) 2) 3) 6)
			C: 42 ± 22.15			C: 16.1 ± 2.78				
Wang et al., (2014)	30/30		NA	41/19		NA	JieDu HuaYu Granule + WM	WM	8 W/48 W	2) 3)
Liu et al., (2015)	30/28		NA	53/5		NA	LiangXue JieDu Huayu Prescriptions + WM	WM	4 W	1) 2) 3)
Duan et al., (2016)	29/29		T: 40 ± 20.02	39/19		T: 14.01 ± 5.31	LiangXue JieDu Huayu Prescriptions + WM	WM	8 W	1)
			C: 41.1 ± 23.31			C: 15.63 ± 6.01				
Zhao, 2016	60/60		T: 45.5 ± 10.5	94/26		NA	JieDu LiangXue Prescriptions + WM	WM	8 W	2) 3)
			C: 45.7 ± 10.7							
Sun et al., (2016)	39/20		T: 16–62	50/9		NA	LiangXue JieDu Huayu Prescriptions + WM	WM	8 W	2) 3)
			C: 20–63							
Xiao et al., (2016)	30/34		NA	NA		NA	QingRe JieDu LiangXue Prescriptions + WM	WM	NA	1) 2) 3) 5)
Liu and Bai., (2017)	45/45		T: 53.32 ± 1.1	47/43		T: 8.15 ± 1.1	JieDu LiangXue Decoction + WM	WM	8 W	1) 2) 3) 5)
			C: 53.22 ± 1.1			C: 8.25 ± 1.2				
Dang et al., (2017)	33/32		T: 45.76 ± 10.65	55/13		T: 14.82 ± 5.46	YinHu TuiHuang Prescriptions + WM	WM	8 W	2) 3) 5)
			C: 46.03 ± 11.12			C: 15.14 ± 6.11				
Lou, 2017	102/54		T:43.45 ± 9.98	129/27		NA	LiangXue JieDu HuaYu Decoction + WM	WM	12 W	1) 2) 3)
			C: 44.19 ± 10.95							
Pang and Yang., (2017)	43/43		T: 36.24 ± 4.69	75/11		T: 2.45 ± 0.21	LiangXue JieDu Decoction + WM	WM	8 W	2) 3)
			C: 35.78 ± 5.01			C: 2.39 ± 0.32				
Shi et al., (2018)	59/59		T: 47.2 ± 7.2	96/22		NA	JieDu HuaYu II Prescriptions + WM	WM	8 W	1) 2) 3)
			C: 48.1 ± 8.1							
Chen et al., (2018)	51/50		NA	53/48		T: 0.2–16	LiangXue JieDu HuaYu Prescriptions + WM	WM	4 W	1) 5)
						C: 0.1–15				
Zhou et al., (2018)	64/56		T: 33–52	72/56		T: 2–15	JieDu JuaYu Granule + WM	WM	8 W	1) 2) 3)
			C: 30–49			C: 2–12				
Yin et al., 2020	49/49		T: 45.17 ± 12.85	82/16		T: 16.3 ± 4.21	LiangXue JieDu HuaYu Decoction + WM	WM	8 W	1) 2) 3) 4) 5)
			C: 44.96 ± 12.34			C: 16.6 ± 4.44				
Shi et al., (2021)	36/39		T: 41.8 ± 12.3	61/14		NA	JieDu LiangXue JianPi Prescriptions + WM	WM	8 W	1) 2)
			C: 43.3 ± 9.6							
Zhang, 2021	48/48		T: 41.27 ± 20.01	78/18		T: 3.58 ± 1.29	LiangXue JieDu HuaYu Decoction + WM	WM	8 W	1) 2) 3) 5)
			C: 41.58 ± 23.29			C: 3.76 ± 1.31				
Huang et al., 2021	40/40		T: 46.5 ± 11.2	50/30		NA	Qinghuang Yin + WM	WM	8 W	1) 2) 3) 4)
			C: 47.8 ± 13.4							

a Outcome measures.

1) total effective rate; 2) liver function; 3) coagulation function; 4) TCM, syndrome score; 5) complications; 6) mortality rate.

T, treatment group; C, control group; NA, not available; WM, western medicine; W, week.

### 3.2 Quality Assessment

**FIGURE 2:**
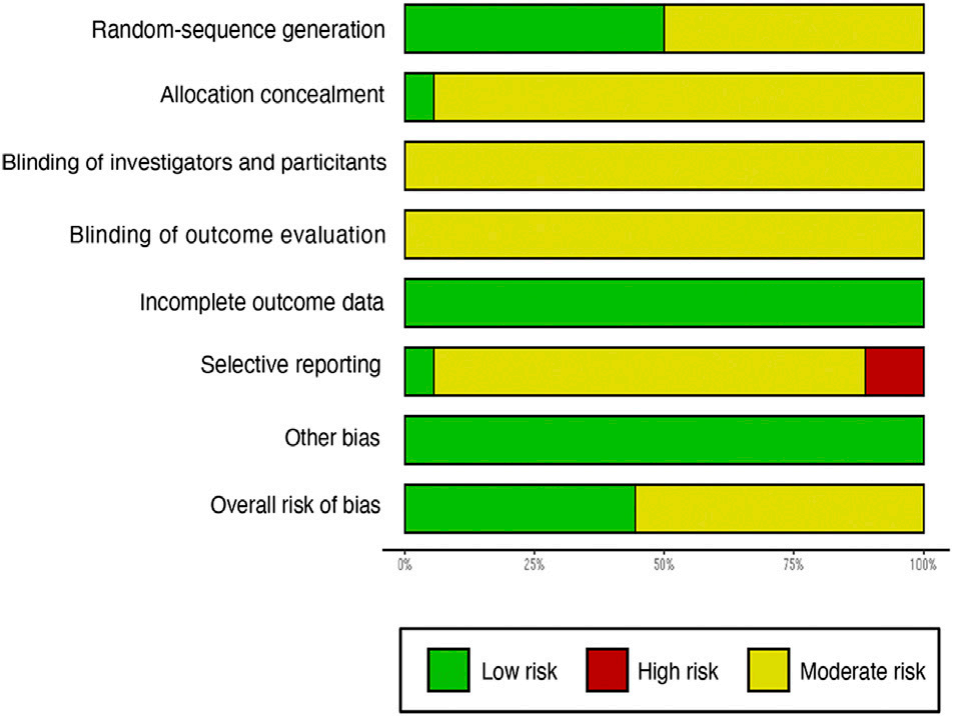
Risk of bias graph.

**FIGURE 3:**
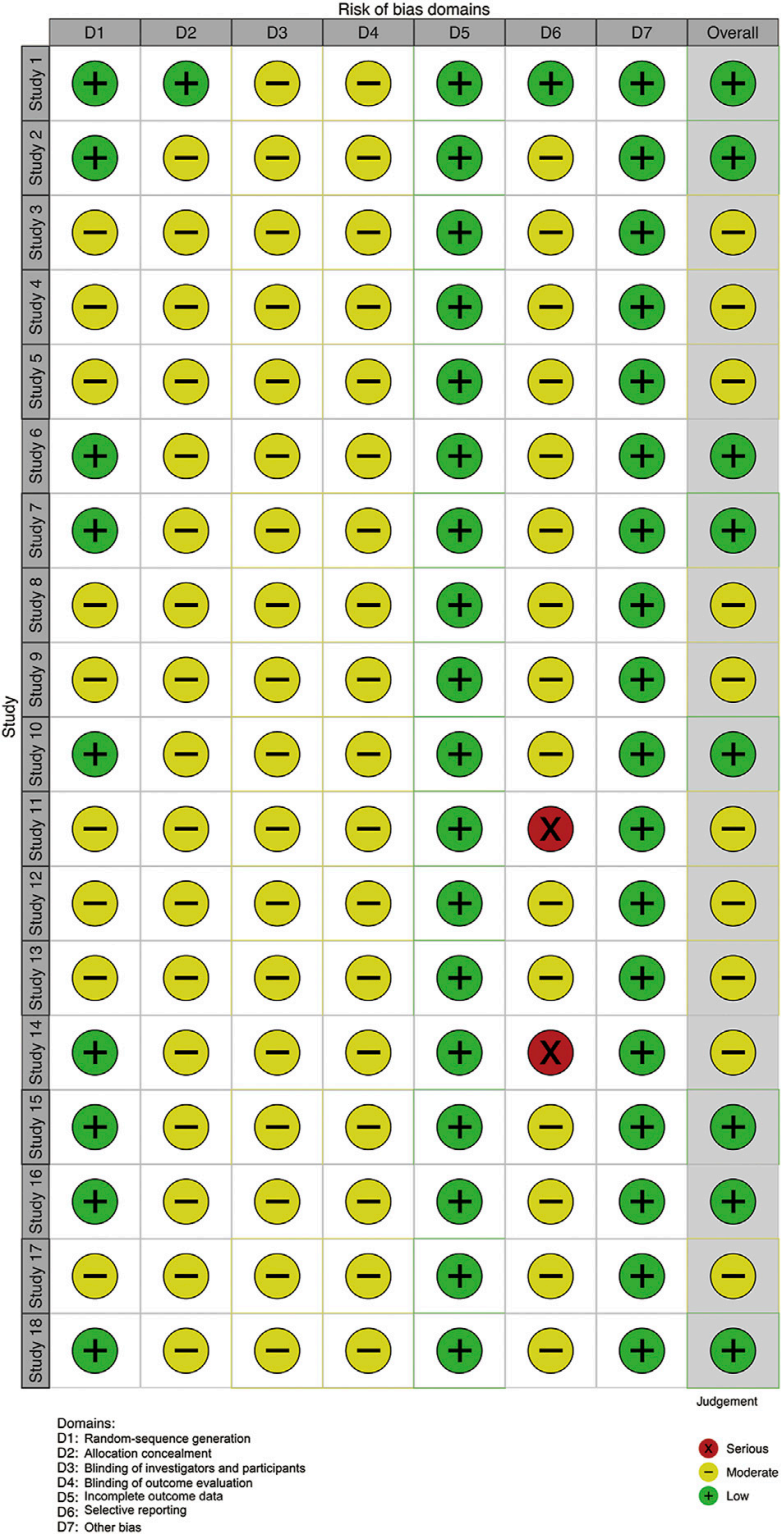
Risk of bias summary. Note: Study 1–18 (Liu et al., 2014; Wang et al., 2014; Liu et al., 2015; Duan et al., 2016; Zhao, 2016; Sun et al., 2016; Xiao et al., 2016; Liu and Bai, 2017; Dang et al., 2017; Lou, 2017; Pang and Yang, 2017; Shi et al., 2018; Chen et al., 2018; Zhou et al., 2018; Yin and Wen, 2018; Shi et al., 2021; Zhang, 2021; Huang et al., 2022).

We evaluated article quality using the bias assessment tool recommended by the Cochrane Collaboration (Figures 2, 3). Nine studies used a random number table, while another nine mentioned the word “random,” but did not describe any randomization methods. With insufficient information to determine the risks of blinding investigators or participants, we classified these studies as having unclear risks. No studies were labeled as having incomplete data or other biases, so they were considered low risk. Four studies reported cases of detachment and provided a reasonable explanation. Overall, the studies included in our meta-analysis were of moderate quality.

### 3.3 Outcome Measures

#### 3.3.1 Total Effective Rate and Mortality Rate

**FIGURE 4:**
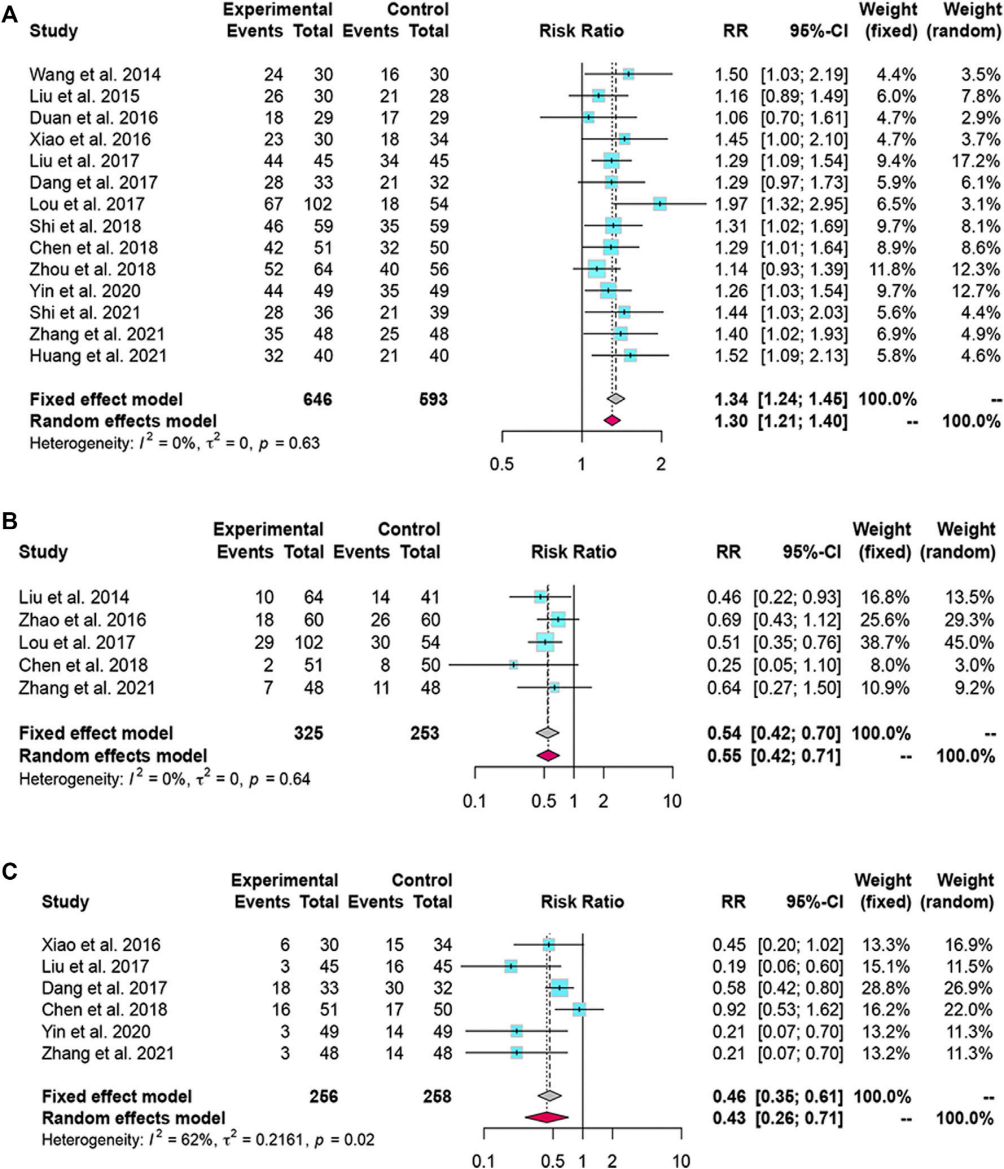
Forest plot for the meta-analysis of total effective rate, mortality rate and complications. **(A)** Forest plot of total effective rate; **(B)** Forest plot of mortality rate; **(C)** Forest plot of complications.

We found 14 and 5 studies that compared the effects of LXJD therapy plus WM on total effective rate and mortality rate, respectively. We adopted a fixed-effects model based on the *p*-value and *I* ^*2*^ value. The RRs (95% CI) were 1.34 (1.24, 1.45) and 0.54 (0.42, 0.70). Compared with WM alone, combination therapy improved effectiveness and reduced mortality (Figures 4A,B).

### 3.4 Complication Rate

Six studies reported complication rates. We selected a random-effects model because the complication rate was heterogeneous (*I* ^*2*^ = 62%, *p* = 0.02). The results suggested that LXJD therapy plus WM had a significantly lower complication rate than WM alone (RR = 0.43; 95% CI: [0.26, 0.71]) (Figure 4C **)**.

### 3.5 Coagulation Function

**FIGURE 5:**
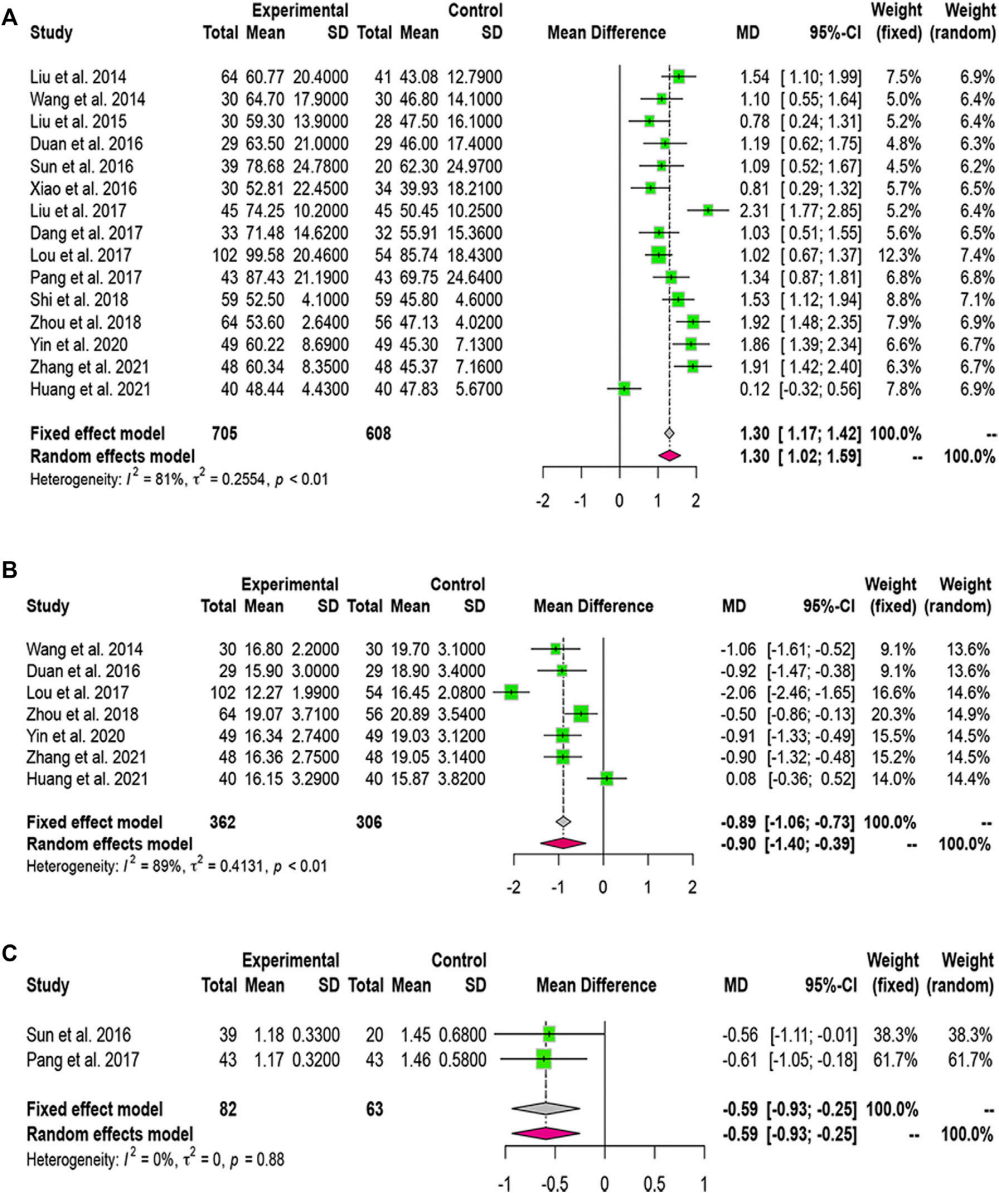
Forest plot for the meta-analysis of coagulation function. **(A)** Forest plot of prothrombin activity; **(B)** Forest plot of prothrombin time; **(C)** Forest plot of international normalized ratio.

Fifteen studies reported PTA and seven studies reported PT data. Based on the *p*-value and *I* ^*2*^ value, we adopted a random-effects model. The MDs (95% CI) of PTA and PT were 1.30 (1.02, 1.59) and -0.90 (−1.40, −0.39), respectively, indicating that PTA levels were significantly higher and PT significantly lower in the combined-treatment group than in the control group (Figures 5A,B).

Two studies included INR data. Heterogeneity analysis led us to use a fixed-effects model. We found that LXJD therapy plus WM significantly reduced the level of INR than WM alone (MD = -0.59; 95% CI: [− 0.93, −0.25]) (Figure 5C).

### 3.6 Liver Function

**FIGURE 6:**
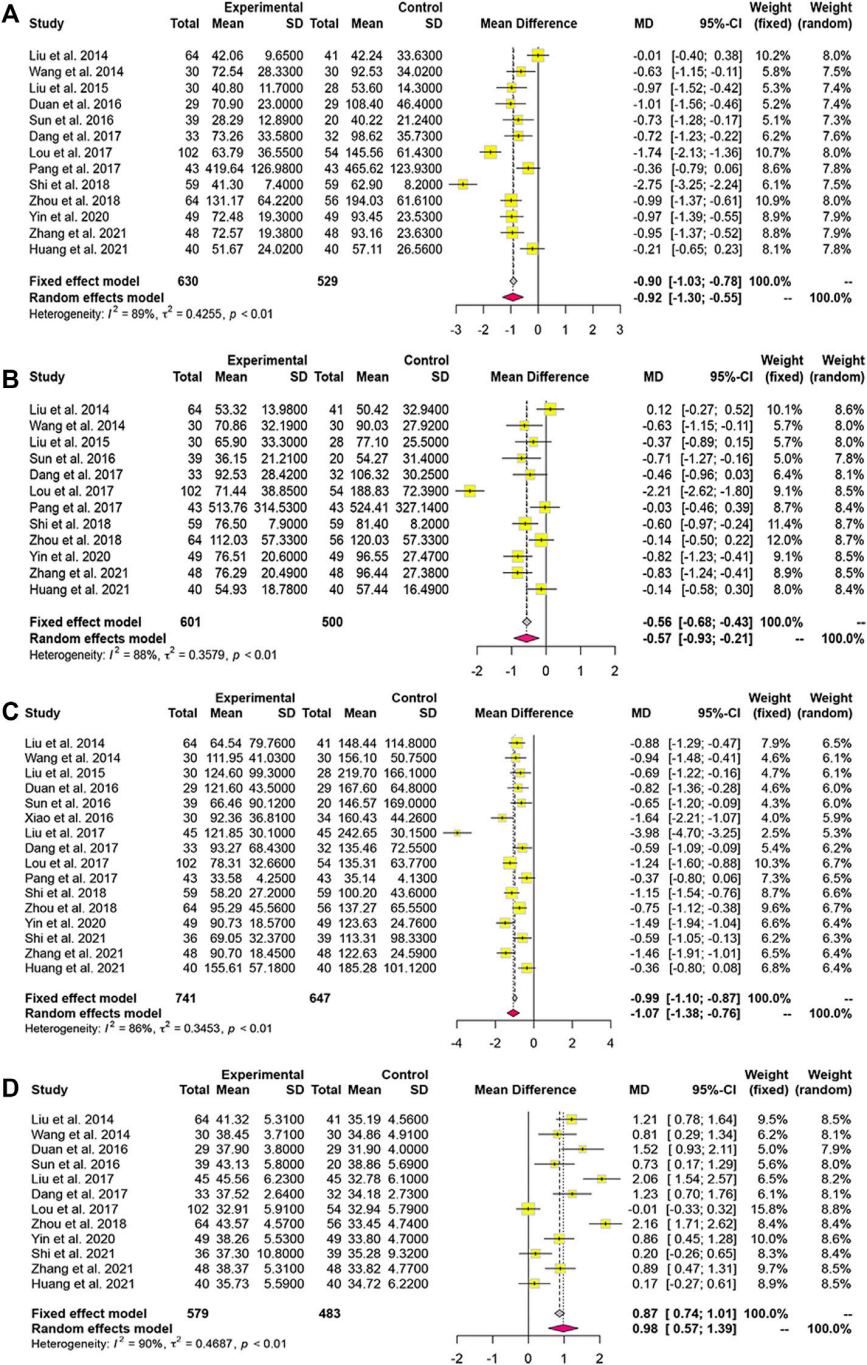
Forest plot for the meta-analysis of liver function. **(A)** Forest plot of alanine aminotransferase; **(B)** Forest plot of aspartate aminotransferase; **(C)** Forest plot of total bilirubin; **(D)** Forest plot of albumin.

Thirteen studies reported serum ALT levels, 12 documented AST and ALB levels, while 16 reported TBIL levels. Based on the *p*-value and *I* ^*2*^ value, we adopted a random-effects model. The MDs (95% CI) of ALT, AST, and TBIL were −0.92 (−1.30, −0.55), −0.57 (−0.93, −0.21), and −1.07 (−1.38, −0.76), respectively (Figures 6A–C). Compared to WM alone, LXJD therapy combined with WM was significantly more effective at decreasing ALT, AST, and TBIL levels. However, the MD (95% CI) of ALB was 0.98 (0.57, 1.39), suggesting that the combined-treatment group did not result in better ALB levels than WM alone (Figure 6D).

### 3.7 Traditional Chinese Medicine Syndrome Score

**FIGURE 7:**
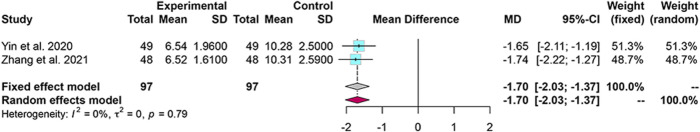
Forest plot for the meta-analysis of TCM syndrome score. TCM: Traditional Chinese medicine.

Two studies reported TCM syndrome scores, and the heterogeneity analysis revealed homogeneity. The fixed-effects model indicated that TCM syndrome score improved more with combined treatment than with WM alone (MD = −1.70; 95% CI: [−2.03, −1.37]) (Figure 7).

### 3.8 Commonly Prescribed Chinese Medicines

We analyzed prescription composition in 18 studies and listed the 10 most frequently used Chinese medicines in LXJD therapy. These were *Artemisia capillaris* Thunb. (Asteraceae), *Paeonia lactiflora* Pall. (Ranunculaceae), *Hedyotis diffusa* Willd. (Rubiaceae), *Salvia miltiorrhiza* Bunge (Lamiaceae), and *Gardenia jasminoides* Ellis (Rubiaceae) (Table 3).

**TABLE 3:** High-frequency Chinese medicines.

Chinese name	English name	Parts of herbs	Counts	Frequency (%)	Picture
Yinchen	*Artemisia capillaris* Thunb. [Asteraceae]	Dried aerial part	17	10.2	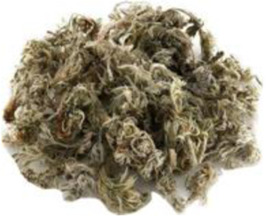
Chishao	*Paeonia lactiflora* Pall. [Ranunculaceae]	Dried root	14	8.4	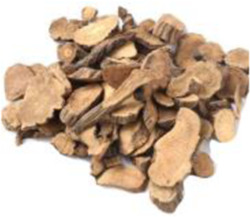
Baihuasheshecao	*Hedyotis diffusa Willd.* [Rubiaceae]	Whole grass	12	7.2	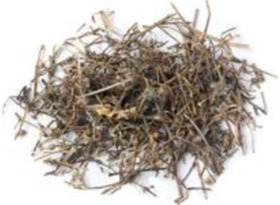
Danshen	*Salvia miltiorrhiza* Bunge [Lamiaceae]	Dried root	12	7.2	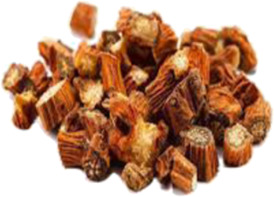
Zhizi	*Gardenia jasminoides* Ellis [Rubiaceae]	Fruit	12	7.2	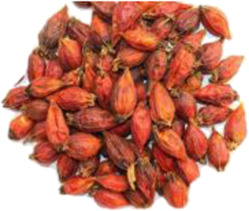
Yujin	*Curcuma aromatica* Salisb. [Zingiberaceae]	Dried root	11	6.6	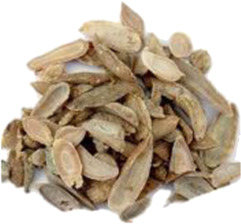
Baizhu	*Atractylodes macrocephala* Koidz. [Asteraceae]	Dried rhizome	9	5.4	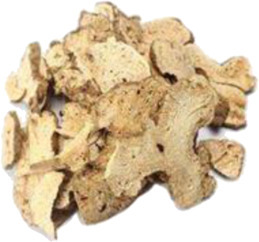
Huangqin	*Scutellaria baicalensis* Georgi [Lamiaceae]	Dried root	8	4.8	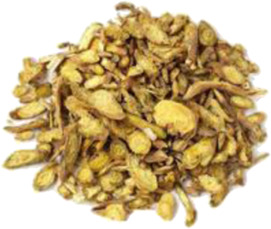
Shengdi	*Rehmannia glutinosa* Libosch. [Scrophulariaceae]	Dried root	7	4.2	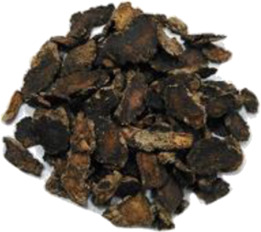
Dahuang	*Rheum palmatum* L. [Polygonaceae]	Dried root	6	3.6	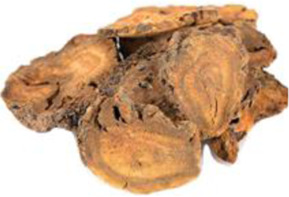

### 3.9 Adverse Events

Adverse outcomes were mentioned in two studies. One (Liu et al., 2014) reported five patients developing nausea after taking TCM. The second (Dang et al., 2017) reported no adverse reactions. These conditions can be significantly relieved through symptomatic treatment. None of the included studies described severe adverse events.

### 3.10 Sensitivity Analysis

Removing each of the included studies did not significantly alter results, indicating that our conclusions had low sensitivity and high stability.

### 3.11 Publication Bias

**FIGURE 8:**
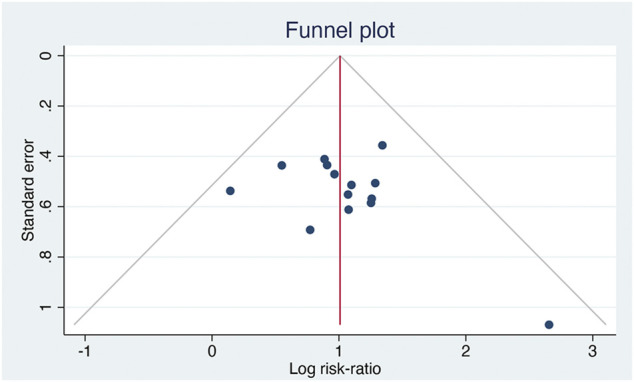
The funnelplot of total effective rate.

Fourteen studies reported the total effective rate. Analysis using inverted funnel plots showed that the distribution was asymmetric, indicating potential publication bias in these studies (Figure 8).

## 4 Discussion

ACLF is a common and severe liver disease with high short-term mortality. Owing to its complex pathogenesis and lack of effective treatments, patients with ACLF tend to seek complementary and alternative therapies. As an auxiliary treatment, TCM has attracted increasing attention and research. Multiple studies have demonstrated that TCM plus WM has various advantages in improving both the prognosis and clinical symptoms of ACLF (Chen et al., 2018; Huang et al., 2022). Contemporary Chinese medicine generally agrees that the core pathogenesis of ACLF is associated with dampness, heat, and pathogens. Therefore, LXJD is an important principle in TCM-based treatment of ACLF (Zhang, 2021).

Here, our meta-analysis confirmed the advantages of LXJD in combination with WM as a treatment for ACLF. The addition of LXJD therapy to WM resulted in higher total effective rate, notably decreasing both mortality and complications, than WM alone. Coagulation and liver function are widely used as therapeutic indicators in clinical practice. Combined treatment significantly lowered PT, INR, ALT, AST, and TBIL levels while increasing PTA levels. These results indicate that LXJD therapy effectively accelerated jaundice elimination and recovery of liver synthesis in patients with ACLF, both key elements to reducing mortality rate. The TCM syndrome score is a common index for evaluating patient recovery. Two studies in our meta-analysis reported this index and demonstrated that adding LXJD therapy significantly reduced TCM syndrome scores compared with WM alone. However, because this analysis contained a small number of studies, further evaluation of TCM syndrome scores is necessary. We also did not find any beneficial effect of LXJD therapy on ALB levels.

The pathobiology of ACLF is characterized by hepatocyte damage, systemic inflammation, and death (Vanlangenakker et al., 2008). Previous clinical, *in vitro*, and *in vivo* studies have reported that TCM has underlying hepatoprotective and pharmacological effects, including anti-inflammatory, antioxidant, anti-apoptotic, and anti-cholestatic effects (Zhuang et al., 2020; Yang et al., 2015; Cai et al., 2020). Specifically, LXJD therapy has multicomponent and multitarget pharmacological effects on the complex pathogenesis of ACLF. For example, paeoniflorin is a main component isolated from *Paeonia lactiflora* Pall. that alleviates inflammatory response, regulates oxidative stress, and protects liver function (Zhang et al., 2015; Chen et al., 2021). Additionally, cryptotanshinone is a major active ingredient of *Salvia miltiorrhiza* Bunge (Lamiaceae) that downregulates inflammatory factors, such as interleukin (IL)-1β, IL-6, and tumor necrosis factor (TNF)-α (Liu et al., 2021). The Qingchangligan formula significantly enhanced liver failure therapy through regulating hepatitis, promoting autophagy, and limiting hepatocyte apoptosis (Zhang et al., 2017). Moreover, the Jidu Liangxue prescription exerted a protective effect against liver failure in mice, *via* a mechanism potentially related to inhibiting the mitochondrial apoptosis signaling pathway (Liu et al., 2022). Network pharmacology and basic research have demonstrated that the Jieduan-Niwan formula downregulates the expression of inflammatory factors, protects against oxidative stress, and inhibits the E2F1-mediated apoptosis signaling pathway to treat ACLF (Liang et al., 2020; Hou et al., 2021).

Our meta-analysis suggested that LXJD therapy causes relatively few adverse events. However, because most included trials did not mention adverse events, we should be cautious in our conclusions regarding the safety of LXJD therapy. None of the studies statistically analyzed differences in adverse events between combined-treatment and control groups. Notably, two studies described adverse side-effects, but they were mild and could be alleviated through symptomatic treatment. No serious adverse events were reported.

This meta-analysis had several limitations. First, although random assignment was mentioned in all of the included studies, only half described a specific randomization method, such as a random number table. Therefore, the findings should be further assessed using high-quality RCTs because study quality may have influenced the chosen indicators (Kjaergard et al., 2001). Second, although LXJD therapy was always used in the combined-treatment group, heterogeneity may nevertheless have been present because herbs and administration courses differed between studies. Third, sample size was relatively small among included studies, with only six involving over 100 patients. These RCTs had different experimental periods ranging from 4 to 12 weeks. Few studies focused on long-term follow-up, and only two followed up at 48 weeks. Fourth, since adverse events were not described in most studies, further evaluation is required to verify the safety of LXJD therapy for ACLF. Finally, all included studies were conducted in China, potentially limiting application to populations in foreign countries.

## 5 Conclusions

Our findings suggest that LXJD therapy combined with WM was more effective than WM alone in treating ACLF, based on improvements to total effective rate, mortality rate, complications, coagulation and liver function, as well as TCM syndrome score. This study provides reliable evidence for clinical practice, showing that LXJD therapy is a promising complementary or alternative treatment for ACLF. However, multicenter, large-sample RCTs with long follow-up periods are needed to better assess the effectiveness and safety of LXJD therapy for ACLF.

## Data Availability

The original contributions presented in the study are included in the article/Supplementary Material, further inquiries can be directed to the corresponding author.
