# Supplementary material for: Evaluation of LiangXue JieDu Therapy in Combination With Western Medicine for Acute-On-Chronic Liver Failure: A Systematic Review and meta-Analysis
Source: Front Pharmacol. 2022 Jul 12;13:905215. doi: 10.3389/fphar.2022.905215 (PMC9315310; doi:10.3389/fphar.2022.905215)
Supplement: Supplementary file 1 [file Table1.DOCX]

**A summary table describing the composition of the preparations.**

**We did not provide chemical analysis because there were no reports in the included studies.**

| **Study** | **Formulation** | **Source** | **Species, concentration** | **Quality**  **control**  **reported?**  **(Y/N)** | **Chemical**  **analysis**  **reported? (Y/N)** |
| --- | --- | --- | --- | --- | --- |
| Liu et al. (2014) | LiangXue JieDu Decoction | Prepared by Liu et al. (2014) | Dried aerial part of *Artemisia capillaris* Thunb.[Asteraceae]30g, Root of Rheum palmatum L. [Polygonaceae]15g, Fruit of Gardenia jasminoides J.Ellis [Rubiaceae]15g, Root of Rehmannia glutinosa Libosch.[Scrophulariaceae]5g, Root of Scutellaria baicalensis Georgi [Lamiaceae]15g, Root of Paeonia lactiflora Pall.[Ranunculaceae]30g, Dried whole grass of Taraxacum mongolicum Hand.-Mazz.[Asteraceae]30g, Root of Curcuma aromatica Salisb.[Zingiberaceae]15g, Root of Salvia miltiorrhiza Bunge [Lamiaceae]15g, Dried root bark of Paeonia suffruticosa Andr.[Ranunculaceae]15g, Root of *Lithospermum erythrorhizon* Sieb. et Zucc.[Boraginaceae]15g, Rhizome of *Atractylodes macrocephala* Koidz.[Asteraceae]15g, and Dry ripe peel of *Citrus reticulata Blanco*[Rutaceae]15g. | N | N |
| Wang et al. (2014) | JieDu HuaYu Granule | Prepared by Wang et al. (2014) | Dried aerial part of *Artemisia capillaris* Thunb.[Asteraceae], Root of *Paeonia lactiflora* Pall.[Ranunculaceae], Root of *Rheum palmatum* L. [Polygonaceae], Whole grass of *Hedyotis diffusa* Willd.[Rubiaceae], Rhizome of *Acorus tatarinowii* Schott[Araceae], and Root of *Curcuma aromatica* Salisb.[Zingiberaceae]. | N | N |
| Liu et al. (2015) | LiangXue JieDu Huayu Prescriptions | Prepared by Liu et al. (2015) | Root of *Paeonia lactiflora* Pall.[Ranunculaceae] 60g, Root of *Salvia miltiorrhiza* Bunge [Lamiaceae]30g, Dried aerial part of *Artemisia capillaris* Thunb.[Asteraceae]30g, Rhizome of *Atractylodes macrocephala* Koidz.[Asteraceae]30g, Root of *Rubia cordifolia* L.[Rubiaceae]30g, Whole grass of *Hedyotis diffusa* Willd.[Rubiaceae]30g, Dry aboveground part of *Siegesbeckia orientalis* L.[Asteraceae]30 g，Tuber of *Bletilla striata*  (Thunb.) Rchb.f. [Orchidaceae]9 g, and Fruit of *Gardenia jasminoides* J. Ellis[Rubiaceae] 9 g | N | N |
| Duan et al. (2016) | LiangXue JieDu Huayu Prescriptions | Prepared by Duan et al. (2016) | Root of Paeonia lactiflora Pall.[Ranunculaceae] 60g, Dried aerial part of *Artemisia capillaris* Thunb.[Asteraceae]  30g, Rhizome of *Atractylodes macrocephala* Koidz.[Asteraceae] 30g, Fruit of *Gardenia jasminoides* J.Ellis [Rubiaceae] 9g，Whole grass of *Hedyotis diffusa* Willd.[Rubiaceae]  30g, Root of *Salvia miltiorrhiza* Bunge [Lamiaceae]30g, Root of *Rubia cordifolia* L.[Rubiaceae] 30g, Dry aboveground part of *Siegesbeckia orientalis* L.[Asteraceae] 30 g，and Tuber of *Bletilla striata*  (Thunb.) Rchb.f. [Orchidaceae] 15g. | N | N |
| Zhao. (2016) | JieDu LiangXue Prescriptions | Prepared by Zhao. (2016) | Dried root bark of *Paeonia suffruticosa* Andr.[Ranunculaceae]15g, Root of *Rehmannia glutinosa* Libosch.[Scrophulariaceae]15g, Root of *Rheum palmatum* L. [Polygonaceae]15g, Root of *Scutellaria baicalensis* Georgi  [Lamiaceae]15g, Root of *Salvia* *miltiorrhiza* Bunge [Lamiaceae]15g, Root of *Lithospermum erythrorhizon* Sieb. et Zucc.[Boraginaceae]15g, Root of *Curcuma aromatica* Salisb.[Zingiberaceae]15g, Fruit of *Gardenia jasminoides* J.Ellis [Rubiaceae]15g, Dry ripe peel of *Citrus reticulata Blanco*[Rutaceae]15g, Rhizome of *Atractylodes macrocephala* Koidz.[Asteraceae]30g, Dried whole grass of *Taraxacum mongolicum* Hand.-Mazz.[Asteraceae]30g, Dried aerial part of *Artemisia capillaris* Thunb.[Asteraceae]30g, and Root of *Paeonia lactiflora* Pall.[Ranunculaceae]30g. | N | N |
| Sun et al. (2016) | LiangXue JieDu Huayu Prescriptions | Prepared by Sun et al. (2016) | Root of *Paeonia lactiflora* Pall.[Ranunculaceae] 60g, Dried aerial part of *Artemisia capillaris* Thunb.[Asteraceae]30g, Fruit of *Gardenia jasminoides* J.Ellis [Rubiaceae] 10g, Whole grass of *Hedyotis diffusa* Willd.[Rubiaceae]30g, Root of *Salvia miltiorrhiza* Bunge [Lamiaceae] 20g, Rhizome of *Atractylodes macrocephala* Koidz.[Asteraceae] 30g, Root of *Rubia cordifolia* L.[Rubiaceae] 15 g, Dry aboveground part of *Siegesbeckia orientalis* L.[Asteraceae] 15g, and Tuber of *Bletilla striata*  (Thunb.) Rchb.f. [Orchidaceae] 15 g | N | N |
| Xiao et al. (2016) | QingRe JieDu LiangXue Prescriptions | Prepared by Xiao et al. (2016) | Root of *Scutellaria baicalensis* Georgi [Lamiaceae]10 g, Root of *Scutellaria baicalensis* Georgi [Lamiaceae]10g, Root of *Gentiana scabra* Bunge [Campanulaceae]10g,Whole grass of *Patrinia scabiosaefolia* Fisch.[Patrinaceae] 20g, Root of *Isatis indigotica* Fortune[Brassicaceae]20g, Dried whole grass of *Taraxacum mongolicum* Hand.-Mazz.[Asteraceae] 30g, Fruit of *Gardenia jasminoides* J. Ellis[Rubiaceae] 60g, Dried aerial part of *Artemisia capillaris* Thunb.[Asteraceae] 30g, Root of *Curcuma aromatica* Salisb.[Zingiberaceae]10g, Whole grass of *Hedyotis diffusa* Willd.[Rubiaceae] 30g, Root of *Scrophularia ningpoensis* Hemsl.[Scrophulariaceae] **15**g, Root of *Rehmannia glutinosa* Libosch.[Scrophulariaceae]30g, and Root of *Glycyrrhiza inflata* Batalin[Fabaceae] 5g. | N | N |
| Liu et al. (2017) | JieDu LiangXue Decoction | Prepared by Liu et al. (2017) | Root of *Rehmannia glutinosa* Libosch.[Scrophulariaceae] **15**g, Root of *Salvia miltiorrhiza* Bunge [Lamiaceae] **15**g, Root of *Scutellaria baicalensis* Georgi [Lamiaceae] 9g, Fruit of *Gardenia jasminoides* J.Ellis [Rubiaceae]  **15**g, Dried whole grass of *Taraxacum mongolicum* Hand.-Mazz.[Asteraceae]30g,Root of *Lithospermum erythrorhizon* Sieb. et Zucc.[Boraginaceae]**15**g, and Root of *Curcuma aromatica* Salisb.[Zingiberaceae] 15 g. | N | N |
| Dang et al. (2017) | YinHu TuiHuang Prescriptions | Prepared by Dang et al. (2017) | Dried aerial part of *Artemisia capillaris* Thunb.[Asteraceae]4**5**g, Rhizome of *Reynoutria japonica* Houtt.[Polygonaceae]**30**g, Root of *Paeonia lactiflora* Pall.[Ranunculaceae] **30**g, Rhizome of *Atractylodes macrocephala* Koidz.[Asteraceae]2**0**g, Root of *Curcuma aromatica* Salisb.[Zingiberaceae]2**0**g, and Seed of *Plantago asiatica* L.[Plantaginaceae]30 g | N | N |
| Lou. (2017) | LiangXue JieDu HuaYu Decoction | Prepared by Lou. (2017) | Root of *Paeonia lactiflora* Pall.[Ranunculaceae]15g, Root of *Rheum palmatum* L. [Polygonaceae]15g, Root of *Daphne genkwa* Sieb. et Zucc.[Thymelaeaceae]15g,  Bark of *Magnolia officinalis* Rehder & E.H.Wilson[Magnoliaceae]15g, Dried root bark of *Paeonia suffruticosa* Andr.[Ranunculaceae]30g, Whole grass of *Patrinia scabiosaefolia* Fisch.[Patrinaceae]30g, Whole grass of *Hedyotis diffusa* Willd.[Rubiaceae]30g, Dried aerial part of *Artemisia capillaris* Thunb.[Asteraceae]20g, Bark of *Phellodendron amurense* Rupr.[Rutaceae]20g, Fruit of *Gardenia jasminoides* J.Ellis [Rubiaceae]20g, Root of *Curcuma aromatica* Salisb.[Zingiberaceae]、Fruit of *Citrus aurantium* L. [Rutaceae]12g, and Root of *Glycyrrhiza inflata* Batalin[Fabaceae] 10g. | N | N |
| Pang et al. (2017) | LiangXue JieDu Decoction | Prepared by Pang et al. (2017) | Root of *Paeonia lactiflora* Pall.[Ranunculaceae]60g, Dried aerial part of *Artemisia capillaris* Thunb.[Asteraceae]30g, Whole grass of *Hedyotis diffusa* Willd.[Rubiaceae]30g, Rhizome of *Atractylodes macrocephala* Koidz.[Asteraceae]30g, Root of *Salvia miltiorrhiza* Bunge [Lamiaceae] 20g, Root of *Rubia cordifolia* L.[Rubiaceae]20g, Dry aboveground part of *Siegesbeckia orientalis* L.[Asteraceae]15g, Tuber of *Bletilla striata*  (Thunb.) Rchb.f. [Orchidaceae]15g, and Fruit of Gardenia jasminoides J.Ellis [Rubiaceae] 10g | N | N |
| Shi et al. (2018) | JieDu HuaYu II Prescriptions | Prepared by Shi et al. (2018) | Dried aerial part of *Artemisia capillaris* Thunb.[Asteraceae]30g, Root of *Paeonia lactiflora* Pall.[Ranunculaceae]50g, Whole grass of *Hedyotis diffusa* Willd.[Rubiaceae]30g, Root of *Rheum palmatum* L. [Polygonaceae]15g, Root of *Curcuma aromatica* Salisb.[Zingiberaceae] 15g, and Rhizome of *Acorus tatarinowii* Schott[Araceae]15g. | N | N |
| Chen et al. (2018) | LiangXue JieDu HuaYu Prescriptions | Prepared by Chen et al. (2018) | Dried aerial part of *Artemisia capillaris* Thunb.[Asteraceae]90g, Root of *Paeonia lactiflora* Pall.[Ranunculaceae]60g, Whole grass of *Hedyotis diffusa* Willd.[Rubiaceae]30g, Root of *Salvia miltiorrhiza* Bunge [Lamiaceae]30g, Rhizome of *Atractylodes macrocephala* Koidz.[Asteraceae]30g, Root of *Rubia cordifolia* L.[Rubiaceae]30g, Dry aboveground part of *Siegesbeckia orientalis* L.[Asteraceae]30g,  Tuber of *Bletilla striata*  (Thunb.) Rchb.f. [Orchidaceae]15g, and Fruit of *Gardenia jasminoides* J.Ellis [Rubiaceae] 12 g | N | N |
| Zhou et al. (2018) | JieDu JuaYu Granule | Prepared by Zhou et al. (2018) | Dried aerial part of *Artemisia capillaris* Thunb.[Asteraceae]30g, Root of *Paeonia lactiflora* Pall.[Ranunculaceae]50g, Root of *Rheum palmatum* L. [Polygonaceae]15g, Whole grass of *Hedyotis diffusa* Willd.[Rubiaceae]  30g, Rhizome of *Acorus tatarinowii* Schott[Araceae]15g, and Root of Curcuma *aromatica Salisb*.[Zingiberaceae]15 g | N | N |
| Yin et al. (2020) | LiangXue JieDu HuaYu Decoction | Prepared by Yin et al. (2020) | Dried aerial part of *Artemisia capillaris* Thunb.[Asteraceae]15g, Root of *Paeonia lactiflora* Pall.[Ranunculaceae]15g, Root of *Salvia miltiorrhiza* Bunge [Lamiaceae]15g, Dried whole grass of *Taraxacum mongolicum* Hand.-Mazz.[Asteraceae]15g, Whole grass of *Hedyotis diffusa* Willd.[Rubiaceae]15g, Root of *Curcuma aromatica* Salisb.[Zingiberaceae]15g, Root of *Rehmannia glutinosa* Libosch.[Scrophulariaceae]20g, Rhizome of *Atractylodes macrocephala* Koidz.[Asteraceae]20g, Fruit of *Gardenia jasminoides* J.Ellis [Rubiaceae]10g, Root of *Scutellaria baicalensis* Georgi [Lamiaceae]10g, Rhizome of *Coptis chinensis* Franch. [Ranunculaceae]10g, and Root of *Lithospermum erythrorhizon* Sieb. et Zucc.[Boraginaceae]10 g | N | N |
| Shi et al. (2021) | JieDu LiangXue JianPi Prescriptions | Prepared by Shi et al. (2021) | Dried aerial part of *Artemisia capillaris* Thunb.[Asteraceae]15g, Fruit of *Gardenia jasminoides* J.Ellis [Rubiaceae]15g, Root of *Scutellaria baicalensis* Georgi [Lamiaceae] 15g, Dried root bark of *Paeonia suffruticosa* Andr.[Ranunculaceae]15g, Root of *Salvia miltiorrhiza* Bunge [Lamiaceae]15g, Rhizome of *Cimicifuga foetida* L.[Ranunculaceae]15g, Root of *Rehmannia glutinosa* Libosch.[Scrophulariaceae] 15g, and Rhizome of *Atractylodes macrocephala* Koidz.[Asteraceae] 15g | N | N |
| Zhang. (2021) | LiangXue JieDu HuaYu Decoction | Prepared by Zhang et al. (2021) | Root of *Curcuma aromatica Salisb.*[Zingiberaceae]15g, Dried aerial part of *Artemisia capillaris* Thunb.[Asteraceae]15g, Root of *Salvia miltiorrhiza* Bunge [Lamiaceae]15g, Root of *Paeonia lactiflora* Pall.[Ranunculaceae]15g, Whole grass of *Hedyotis diffusa* Willd.[Rubiaceae]15g, Dried whole grass of *Taraxacum mongolicum* Hand.-Mazz.[Asteraceae]15g, Rhizome of *Atractylodes macrocephala* Koidz.[Asteraceae]20g, Root of *Rehmannia glutinosa* Libosch.[Scrophulariaceae]20g, Fruit of *Gardenia jasminoides* J.Ellis [Rubiaceae]10g, Root of *Lithospermum erythrorhizon* Sieb. et Zucc.[Boraginaceae]10g, Rhizome of *Coptis chinensis* Franch. [Ranunculaceae]10g, and Root of *Scutellaria baicalensis* Georgi [Lamiaceae]10g | N | N |
| Huang et al. (2021) | Qinghuang Yin | Prepared by Huang et al. (2021) | Dried aerial part of *Artemisia capillaris* Thunb.[Asteraceae] 30g, Root of *Paeonia lactiflora* Pall.[Ranunculaceae] 20g, Root of *Salvia miltiorrhiza* Bunge [Lamiaceae] 30g,  Whole grass of *Lysimachia christinae* Hance[Primulaceae]30g, Root of *Isatis indigotica* Fortune[Brassicaceae] 30g, Fruit of *Gardenia jasminoides* J.Ellis [Rubiaceae] 30g, Root of *Scutellaria baicalensis* Georgi [Lamiaceae] 15g, Root of *Bupleurum chinense* DC.[Apiaceae]  15g, Seed of *Plantago asiatica* L.[Plantaginaceae]15g, Root of *Rheum palmatum* L. [Polygonaceae]15g, and  Root of *Glycyrrhiza inflata* Batalin[Fabaceae] 6g | N | N |
